# Heterologous expression and characterization of a malathion-hydrolyzing carboxylesterase from a thermophilic bacterium, *Alicyclobacillus tengchongensis*

**DOI:** 10.1007/s10529-013-1195-5

**Published:** 2013-06-26

**Authors:** Zhenrong Xie, Bo Xu, Junmei Ding, Lingyun Liu, Xuelin Zhang, Junjun Li, Zunxi Huang

**Affiliations:** 1 Engineering Research Center of Sustainable Development and Utilization of Biomass Energy, Ministry of Education, Kunming, Yunnan 650500 People’s Republic of China; 2 College of Life Sciences, Yunnan Normal University, Kunming, Yunnan 650500 People’s Republic of China

**Keywords:** Carboxylesterase, Degradation, Expression, Malathion

## Abstract

A carboxylesterase gene from thermophilic bacterium, *Alicyclobacillus tengchongensis*, was cloned and expressed in *Escherichia coli* BL21 (DE3). The gene coded for a 513 amino acid protein with a calculated molecular mass of 57.82 kDa. The deduced amino acid sequence had structural features highly conserved among serine hydrolases, including Ser204, Glu325, and His415 as a catalytic triad, as well as type-B carboxylesterase serine active site (FGGDPENITIGGQSAG) and type-B carboxylesterase signature 2 (EDCLYLNIWTP). The purified enzyme exhibited optimum activity with β-naphthyl acetate at 60 °C and pH 7 as well as stability at 25 °C and pH 7. One unit of the enzyme hydrolyzed 5 mg malathion l^−1^ by 50 % within 25 min and 89 % within 100 min. The enzyme strongly degraded malathion and has a potential use for the detoxification of malathion residues.

## Introduction

Malathion [*S*-(1,2-dicarbethoxyethyl)-*O*,*O*-dimethyldithiophosphate; also known as carbophos, maldison, and mercaptothion] is a non-systemic, wide-spectrum organophosphorus pesticide used for public health, residential, and agricultural purposes. However, malathion is toxic to living organisms including humans because it is easily absorbed by the gastrointestinal tract, skin, mucous membranes, and lungs (Inderjeet et al. 1997; Kumar et al. 2010). Malathion targets the central nervous and immune system, thereby affecting many organs and functions. These findings reveal that malathion is potentially harmful to human health and the ecosystem. Therefore, efficient strategies must be urgently developed to solve these problems caused by malathion residues.

Biodegradation is an important environment biotechnology for the treatment of organic pollutants. One treatment strategy is the use of some key enzymes to break down pesticide residues. Carboxylesterases (carboxylic ester hydrolase; EC 3.1.1.1) can degrade organophosphorus pesticides, carbamates, pyrethoid insecticides, and organic chloride pesticides (Vontas et al. 2000; Zhang et al. 2004; Barata et al. 2004; Nishi et al. 2006). Carboxylesterases can also catalyze the degradation of malathion to the detoxication products monoacid and diacid derivatives (Yoshii et al. 2007a).

Some carboxylesterases from animals, plants, and insects have been purified, characterized and used to degrade malathion (Miller et al. 1999; Yoshii et al. 2007b; Cao et al. 2008). Several malathion-degrading strains have been isolated, and degradation studies have also been carried out (Foster and Bia 2004; Hashmi et al. 2004; Goda et al. 2010). Microbial carboxylesterase genes have been cloned and expressed as well (Prim et al. 2001; Ewis et al. 2004), but studies on the use of microbial carboxylesterases for pesticide degradation are limited.

Next-generation sequencing technologies enable high-throughput functional genomic research. For example, a novel xylanase from the genome sequence of *Thermonanarobacterium saccharolyticum* NTOU1 (Hung et al. 2011) and pectate lyase from the genome sequence of *Bacillus* sp. I4 (Zhou et al. 2012) have been cloned and expressed in *Escherichia coli* BL21 (DE3). However, a carboxylesterase from a thermophilic bacterium, *A. tengchongensis*, for malathion degradation has not yet been heterologously expressed. In the present study, we cloned and expressed a carboxylesterase (D1CarE5) from the genomic DNA of this thermophile whose whole genome has been sequenced with a Solexa Genome Analyzer. We have thus gained insight into the characteristics of recombinant D1CarE5 and its malathion-degradation potential.

## Materials and methods

### Strains and reagents

*Alicyclobacillus tengchongensis* was isolated from a hot spring in Tengchong, Yunnan, China. *Escherichia coli* BL21 (DE3) and the expression vector, pET28a(+), were from Novagen. Malathion (99 % purity) was from Sigma. Taq DNA polymerase, Pyrobest NDA polymerase, dNTPs, *Bam*HI and *Xho*I were from TaKaRa. Genomic DNA isolation, DNA purification, and plasmid isolation kits were from Tiangen. All other chemicals were analytical grade.

### Sequence analyses

Genomic DNA of the thermophilic *A. tengchongensis* strain was extracted using a Tiangen genomic DNA isolation kit from cells grown overnight at 50 °C. Genome sequencing was performed by the Beijing Genomics Institute (Guangzhou, China) using a Solexa Genome Analyzer, and a partial genomic sequence was obtained. The full-length carboxylesterase gene *D1CarE5* was revealed based on the prediction of ORFs from a partial genomic sequence by the GeneMark.hmm online tool (version 2.8; http://exon.gatech.edu/GeneMark/gmhmm2_prok.cgi). The signal peptide in the amino acid sequence (D1CarE5) deduced from *D1CarE5* was predicted using SignalP (http://www.cbs.dtu.dk/services/SignalP/). The identity values of the protein sequences were obtained from the online BLASTP program (http://www.ncbi.nlm.nih.gov/BLAST/). Defined structural features were analyzed using the Prosite Database at ExPASy (http://au.expasy.org/prosite/). Phylogenetic tree was constructed using MEGA 4.1 software by neighbor-joining method. Other sequence analyses were performed using BioXM 2.6 software (Nanjing Agricultural University, Nanjing, China).

### Expression of *D1CarE5* gene

The nucleotide sequence of putative *D1CarE5* ORF from *A. tengchongensis* was cloned by PCR. The forward primer with a *Bam*HI restriction site was 5′-CGCGGATCCATGCAAAGCATGCTGCGG-3′, and the reverse primer with a *Xho*I restriction site was 5′-CCGCTCGAGGAAAATGTACTGTTTTTGCTTAAAG-3′. The PCR conditions were as follows: 5 min of denaturation at 94 °C, 30 cycles of denaturing at 94 °C for 45 s, primer annealing at 50 °C for 30 s, extension at 72 °C for 1 min, and a final extension at 72 °C for 5 min. The PCR products were purified using a PCR purification kit (TianGen). The pET28a (+) vector and PCR products were completely digested with *Bam*HI and *Xho*I, respectively. The recombinant plasmid was transformed into *E. coli* BL21 (DE3) for protein expression. The transformed strains were grown in LB medium containing 50 μg kanamycin ml^−1^ at 37 °C until the OD_600_ reached 0.3. Protein expression was induced by adding IPTG to 0.05 mM, the culture was shaken for 20 h at 20 °C, and the cells were harvested by centrifugation.

### Purification of recombinant D1CarE5

Cells were harvested by centrifugation at 10,000×*g* for 10 min at 4 °C, washed with sterile distilled H_2_O, and resuspended in sterilized ice-cold buffer A (20 mM Tris/HCl and 0.5 M NaCl; pH 7.2). The cells were disrupted by sonication (7 s, 150 W) on ice for several times and centrifuged at 10,000×*g* for 10 min at 4 °C. The supernatant was applied to a Ni^2+^-NTA agarose gel column for purification with a linear imidazole gradient of 20–500 mM in buffer A.

The purified protein was detected by PAGE using a 5 % (v/v) stacking gel and a 12 % (v/v) resolving gel. Protein concentration was determined by Bradford’s method using bovine serum albumin as the standard.

### Carboxylesterase assay

D1CarE5 activity was assayed with β-naphthyl acetate as substrate by the method of Van (Van 1962). Absorbance was measured at 554 nm for β-naphthyl acetate, and one unit of activity (U) was defined as the amount of enzyme that produced 1 μmol β-naphthol from the substrate per min. The *K*_m_ and *V*_max_ values for the purified D1CarE5 were determined using 0.1–0.95 mM β-naphthyl acetate as substrate at 60 °C in potassium phosphate buffer (pH 7).

### Nucleotide sequence accession number

The nucleotide sequences for 16S rDNA and *D1CarE5* of the thermophilic *A. tengchongensis* strain were deposited in GenBank under accession numbers DQ351931 and JX101458, respectively.

### Pesticide degradation and analytical methods

Malathion degradation by D1CarE5 was determined by the method of Leng and Qiao (1986) with some modifications. Malathion was dissolved in acetone (100 mg/l) and 300 μl was mixed with 4.7 ml 25 mM potassium phosphate buffer (pH 7) and 1 ml diluted enzyme (1 U/ml). The total reaction volume was 6 ml. Malathion solution without enzyme served as a control. The mixtures were incubated at 37 °C, 0.5 ml samples were collected at intervals, and 0.5 ml *n*-hexane was added to extract the product. The supernatant was collected by centrifugation and dried over anhydrous Na_2_SO_4_. The residual malathion was analyzed using a GC/MS system equipped an FID detector with a DB-17 column (30 m × 0.32 mm × 0.25 μm). The chromatographic conditions for detecting malathion were as follows: no split ratio; injection volume, 1 μl; injector program, 205 °C initially for 2.5 min and then increasing to 220 °C for 2 min at 10 °C min^−1^. Different concentration gradients of a standard malathion solution were also injected to construct a standard curve.

## Results and discussion

### Sequence analyses

**Fig. 1:**
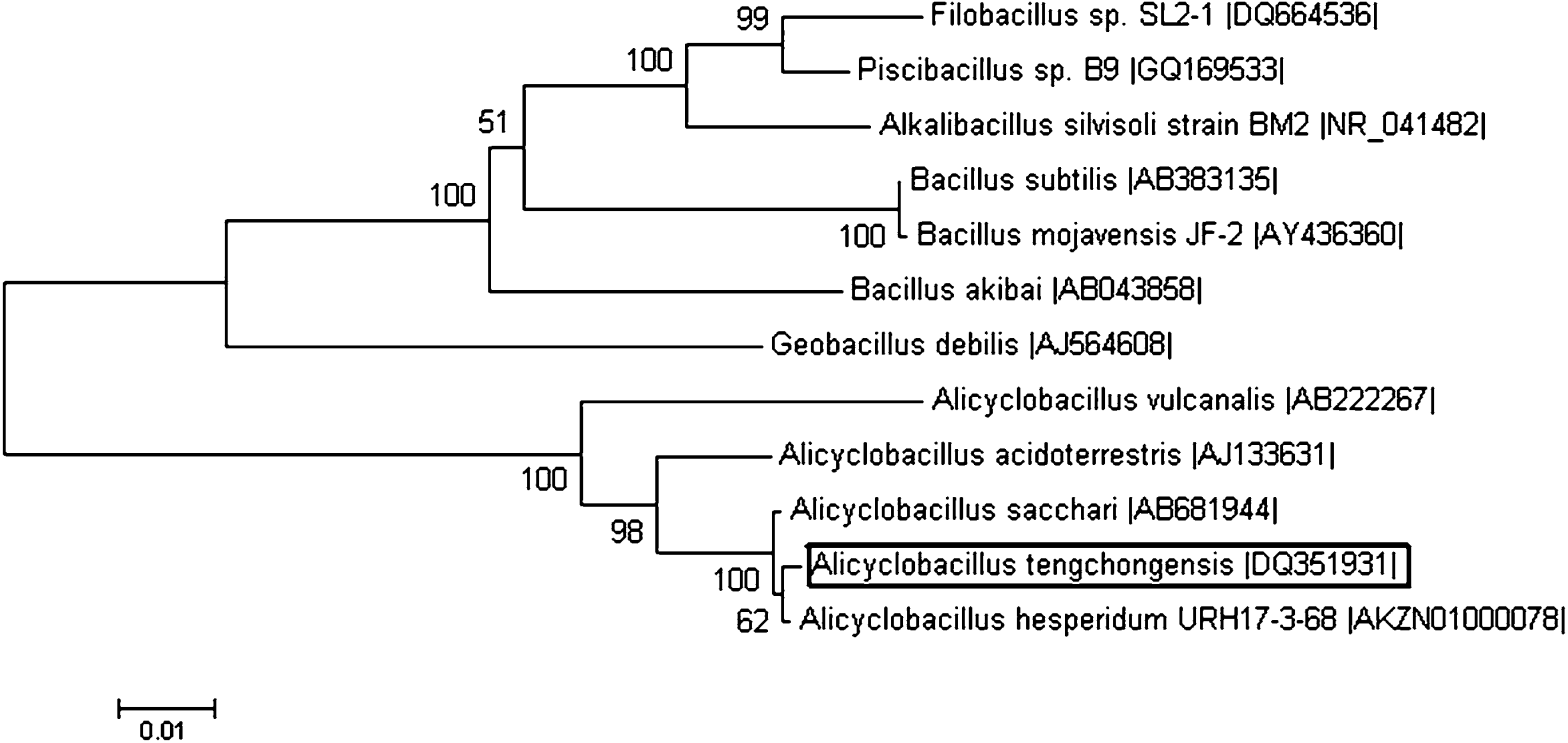
The 16S rDNA-based neighbor-joining tree showing the phylogenetic position of *Alicyclobacillus tengchongensis* strain. Boostrap values (*n* = 1,000 replicates) are reported as percentages (only values above 50 % are given). Sequences were obtained from GenBank (http://www.ncbi.nlm.nih.gov). The *scale bar* represents the number of changes per nucleotide position. Accession numbers are given at the end of each species name. The strain in the present study is shown in *black bars*

A 1,542 bp-long ORF that encoded for a 513 amino acid protein was found. This ORF had a predicted molecular weight (Mr) of 57.82 kDa and pI of 4.52. No signal sequence was found. The deduced amino acid sequence of D1CarE5 showed homology to type-B carboxylesterases. Comparison of the derived amino acid sequence of D1CarE5 with those in the GenBank database showed that it had 33, 36, 68, and 71 % identity to the putative type-B carboxylesterase from *Candidatus Solibacter usitatus* Ellin6076 (accession number: YP_823360), *Emticicia oligotrophica* DSM 17448 (accession number: YP_006874330), *Paenibacillus lactis* 154 (accession number: ZP_09003155), and *Thermobacillus composti* KWC4 (accession number: YP_007213916), respectively. Meanwhile, D1CarE5 shared 97 % identity with the putative carboxylesterase from genome sequence of *Alicyclobacillus hesperidum* URH17-3-68 in GenBank that has not been reviewed and whose function is unknown (Wang et al. 2012; accession number: ZP_10953092). Comparison of the 16S rDNA sequences and phylogenetic analysis showed that the high identity can be attributed to the fact that the two strains belong to the same genus (Fig. 1). In addition, several microbial carboxylesterase genes have been cloned and expressed (Prim et al. 2001; Ewis et al. 2004), but the highest identity is only 34 %. Moreover, the association of these genes with malathion degradation has not been reported.

**Fig. 2:**
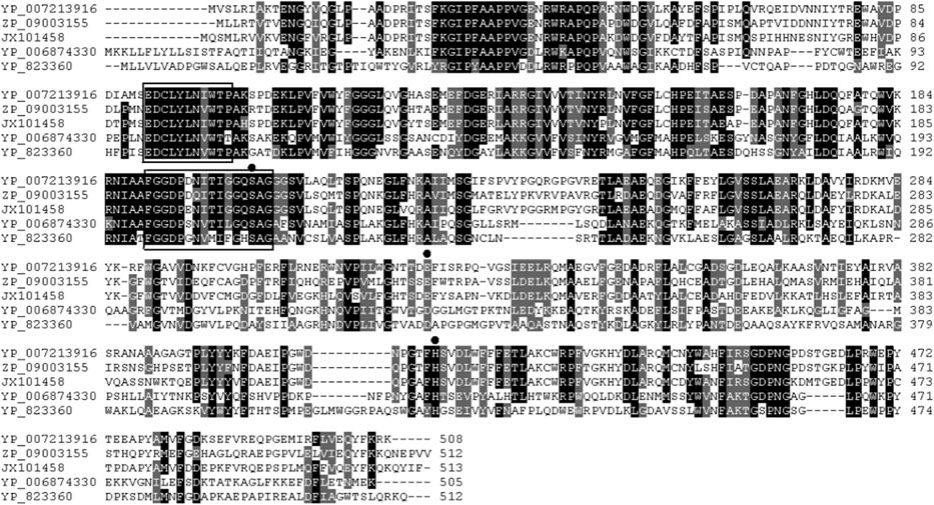
Amino acid sequence alignment of D1CarE5 of type-B carboxylesterases. D1CarE5 showed 33–71 % sequence identity with type-B carboxylesterases from *Candidatus Solibacter usitatus* Ellin6076, *Emticicia oligotrophica* DSM 17448, *Paenibacillus lactis* 154, *Thermobacillus composti* KWC4, and D1CarE5 from a thermophilic *Alicylobacillus tengchongensis* strain (GenBank accession number: JX101458; this study). The catalytic triad residues S (Ser), E (Glu)/D (Asp), H (His) are indicated by* black dots*. Both type-B carboxylesterase serine active site and type-B carboxylesterase signature 2 are *underlined* with *black bars*

D1CarE5 was characterized by a type-B carboxylesterase signature 2 motif (EDCLYLNIWTP) at positions 92–102, another type-B carboxylesterase serine motif (FGGDPENITIGGQSAG) at positions 191–206, and notably the catalytic triad comprising Ser204, Glu325, and His415, which may form a charge-relay system (Gopalapillai et al. 2005). The sequence Gly-Xaa-Ser-Xaa-Gly (Gly202-Gln203-Ser204-Ala205-Gly206) was conserved in all carboxylesterases (Fig. 2).

### Expression and purification of D1CarE5

**Fig. 3:**
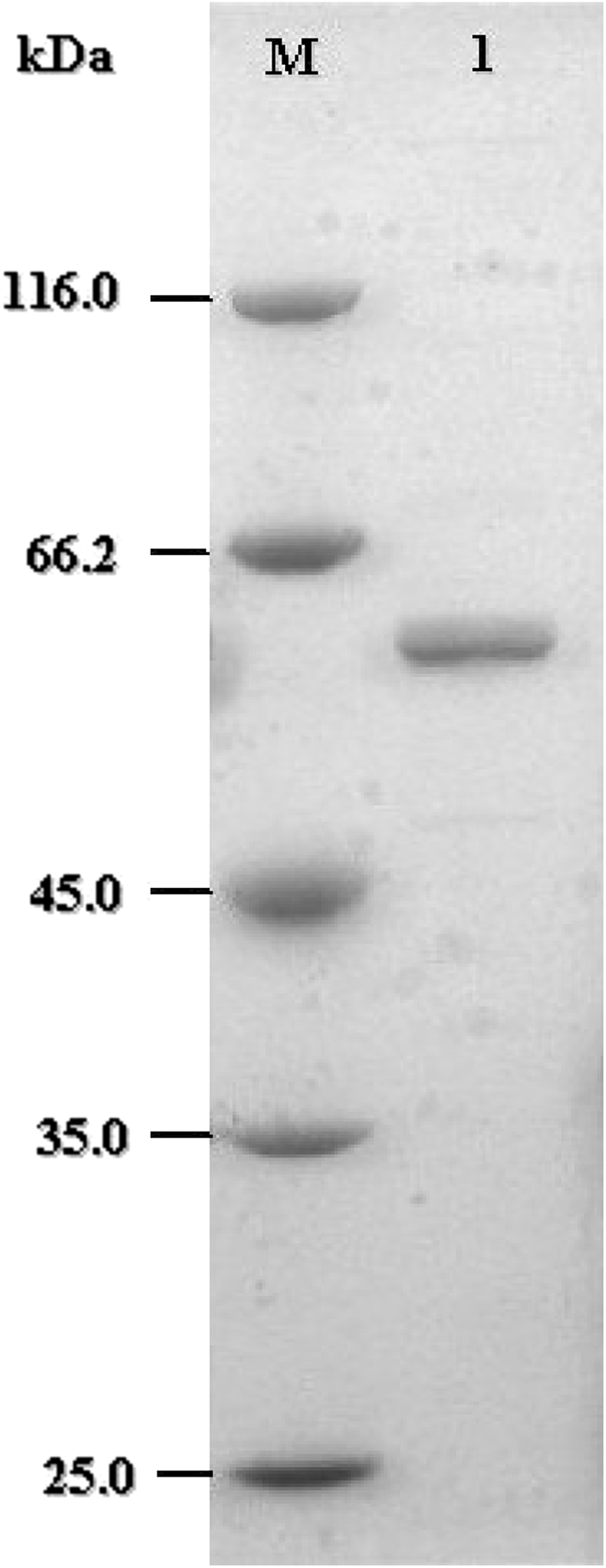
SDS-PAGE analyses of D1CarE5. *Lane* M, low-molecular weight markers; 1, D1CarE5 purified by Ni^2+^-NTA chelating affinity chromatography

*D1CarE5* gene cloned into pET28a (+) was used to transform *E. coli* BL21 (DE3) for expression and induced with 0.5 mM IPTG at 20 °C for 20 h. The crude enzyme extracted from recombinant *E. coli* BL21 (DE3) cells was purified to electrophoretic homogeneity by Ni^2+^-NTA metal chelating affinity chromatography (Fig. 3). The purified enzyme with His_6_ migrated as a single band on SDS-PAGE with a molecular mass of ~58 kDa, which was close to the calculated value of 57.82 kDa. The molecular mass of D1CarE5 differed from previous reports on pesticide-degrading enzymes, such as type-B carboxylesterase (53 kDa) from *Bacillus* sp. BP-7 (Prim et al. 2001), carboxylesterase (60 kDa) from mouse liver microsomes (Stok et al. 2004), carboxylesterase B1 (65 kDa) from *Culexpipiens* (Lan et al. 2006), and carboxylesterase (31 kDa) from *Sphingobium* sp. strain JZ-1 (Wang et al. 2009).

### Enzyme characterization

**Fig. 4:**
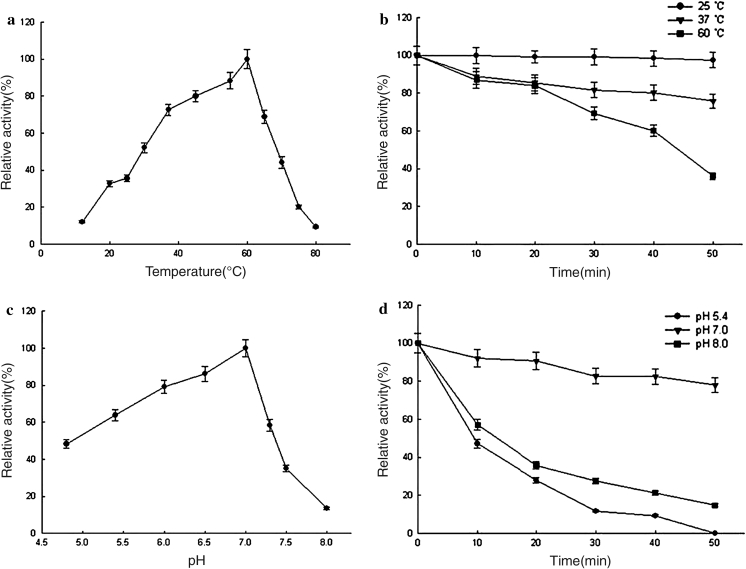
The characterization of purified D1CarE5. **a** Effect of temperature on D1CarE5 activity measured in potassium phosphate buffer (pH 7) at 12–80 °C. **b** Thermostability assay. Purified D1CarE5 was pre-incubated in potassium phosphate buffer (pH 7) at 25, 37, or 60 °C. Aliquots were removed at specific time points for the measurement of residual activity at 37 °C. **c** Effect of pH on D1CarE5 activity. The enzyme activity was determined at 37 °C from pH 4.8 to 8. **d** pH stability assay. After pre-incubation of the enzyme at pH 5.4, 7, or 8, aliquots were removed at specific time points for the measurement of residual activity at 37 °C. The residual activity was expressed as a percentage of the activity measured at 37 °C in potassium phosphate buffer (pH 7). The *error bars* represent the mean ± standard deviation (*n* = 3)

The optimum temperature for D1CarE5 activity was 60 °C (Fig. 4a). The enzyme was stable at 25 °C, and >75 % activity remained at 37 °C; by contrast, only 36 % activity remained at 60 °C after 50 min (Fig. 4b). The purified enzyme was optimally active at pH 7 (Fig. 4c) and retained >70 % of its initial activity after incubation in pH 7 buffer for 50 min at 37 °C (Fig. 4d); by contrast, the enzyme was not stable at pH 5.4 or 8. The activity of purified D1CarE5 was also determined in the presence of different metal ions and chemical reagents (data not shown). Enzyme activity was strongly inhibited by 1 mM Zn^2+^, Hg^2+^, Cu^2+^, Ag^+^, and 1 % (w/v) SDS, whereas Pb^2+^ and Mg^2+^ activated D1CarE5. The addition of 1 % (v/v) acetone and 1 % (v/v) ethanol had little effect on enzyme activity. The *K*_m_ for β-naphthyl acetate was 1 mM, and *V*_max_ was 3.2 μmol min^−1^ mg^−1^ (data not shown).

### Degradation of pesticides by the recombinant D1CarE5

**Fig. 5:**
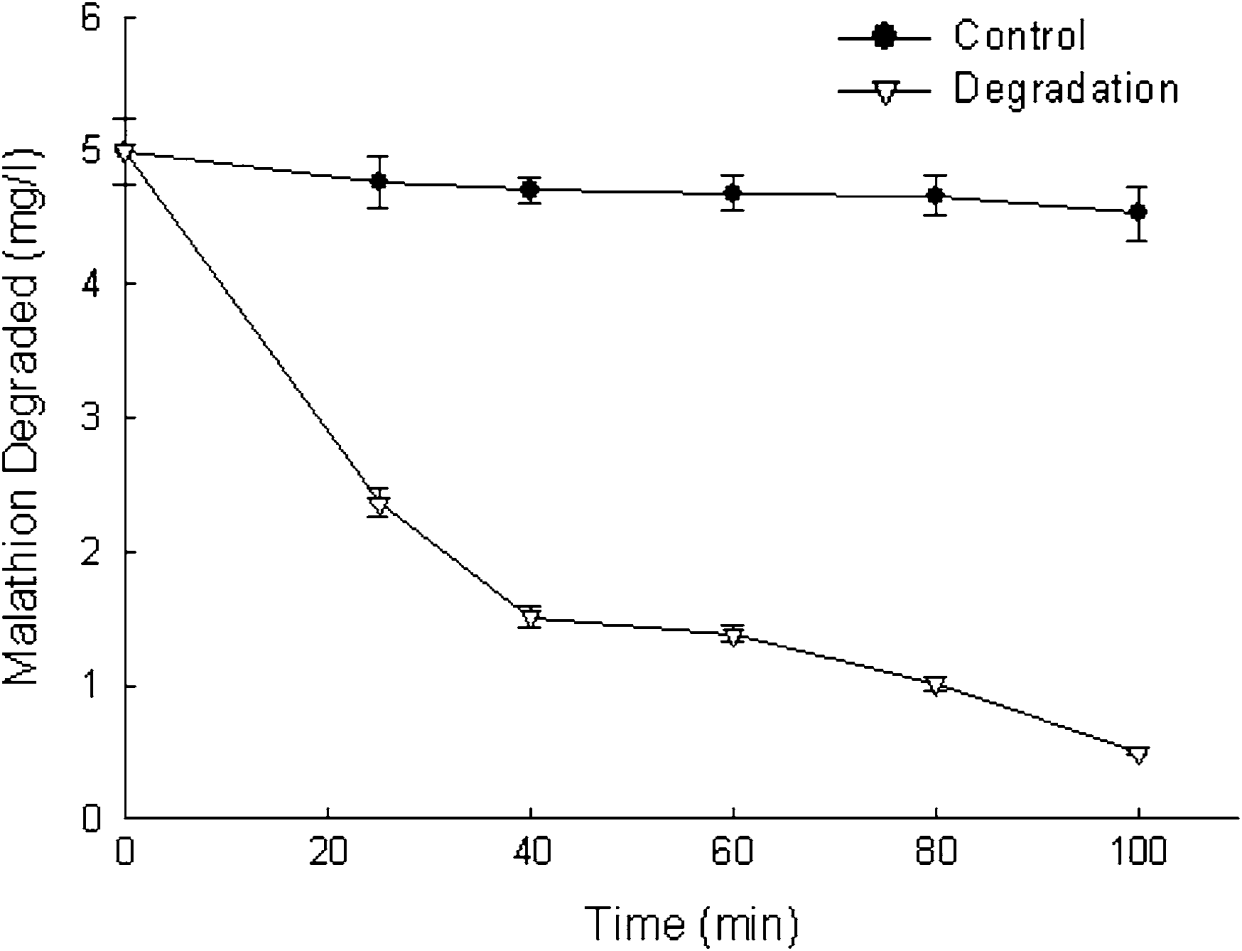
Malathion degradation by purified D1CarE5. The reaction was performed in a 6 ml graduated tube containing 1 U enzyme, 5 mg malathion l^−1^, and potassium phosphate buffer (25 mM, pH 7) at 37 °C. A malathion solution without enzyme served as a control. The *error bars* represent the mean ± SD (*n* = 3)

Malathion degradation by purified D1CarE5 is shown in Fig. 5. The recombinant D1CarE5 (1 U/ml) hydrolyzed 50 % malathion (5 mg/l) within 25 min and 89 % malathion within 100 min. Although a number of bacteria with malathion hydrolase activity have been investigated (Foster and Bia 2004; Hashmi et al. 2004; Goda et al. 2010; Baljinder et al. 2012), recombinant carboxylesterases for malathion degradation have not been reported in *Alicyclobacillus*. The enzyme also shows stronger malathion degradation than carboxylesterase E4 from peach-potato aphid (Lan et al. 2005).

## Conclusions

We cloned and expressed *D1CarE5* gene, purified the recombinant carboxylesterase and gained insight into the characteristics and malathion degradation by the recombinant D1CarE5. This is the first report on the heterologous expression of a carboxylesterase from *Alicyclobacillus* for malathion degradation. The culture supernatant of the bacterium was unable to degrade malathion and carboxylesterase activity was not detected (data not shown). The results indicated that the proposed strategic approach based on the microbial genome was an efficient and rapid method of finding functional carboxylesterases. Our work also showed that the recombinant enzyme has potential use for degrading environmental malathion. Further studies on the degradation of other pesticides by recombinant D1CarE5 and on the underlying mechanisms are underway.
